# Frequency of Private Prayer Predicts Survival Over 6 Years in a Nationwide U.S. Sample of Individuals with a Chronic Illness

**DOI:** 10.1007/s10943-023-01870-z

**Published:** 2023-07-29

**Authors:** Gail Ironson, Salman Shaheen Ahmad

**Affiliations:** 1 Departments of Psychology and Psychiatry, University of Miami, Coral Gables, USA; 2 Department of Psychology, University of Miami, 5665 Ponce de Leon Blvd, Coral Gables, FL 33124-0751 USA

**Keywords:** Prayer, Religion, Spirituality, Mortality, Health

## Abstract

Prayer is central to religious/spiritual life, and there are many claims for its effectiveness. However, few studies have examined whether frequency of prayer predicts survival in people with a chronic illness. This study follows a nationwide United States sample of people with a chronic illness (*N* = 1931) from 2014 to 2020. Those who prayed on a daily basis or more were significantly more likely to survive over 6 years (Hazard Ratio = 1.48; 95% Confidence Intervals: 1.08–2.03) compared with those who prayed less often, after controlling for biomedical variables (age, medical symptoms), sociodemographics (sex, race, and education), psychosocial variables (depression, social support), and health behaviors (alcohol use, smoking, exercise, and body mass index/weight).

## Introduction

Individuals often turn to religion in times of crises. After 9/11, 90% of people turned to religion/God to cope (Schuster et al., 2001). Medical illness is one such crisis, and during the COVID-19 pandemic, around 24% of adults in the United States (U.S.) stated that their faith had become stronger, and online searches for the word “prayer” were at their highest level ever recorded in over 90 countries (Bentzen, 2021). Prayer is one of the most essential aspects of religion, and one of the most widely practiced spiritual rituals for people in the U.S. In a survey of over 35,000 people in the U.S., 55% reported praying at least daily, and only 23% reported that they pray seldom or never (Pew, 2022). McCaffrey et al. (2004) found that 35% of respondents in a national survey used prayer for health concerns, and Barnes et al. (2002) found that prayer for oneself (43%) and for others (24.4%) were the most commonly used “alternative medicine.”

Among individuals with chronic medical conditions, spirituality and religiousness are associated with fewer depressive symptoms (e.g., Lucette et al., 2016). While meta-analyses on religion/spirituality generally show a 20–25% reduction in mortality (Chida et al., 2009), especially in initially healthy people, the majority of these studies used church attendance as the predictor, effects for those who were ill were much weaker, and prayer was not separated out as a predictor. And while books on the effects of prayer are quite popular, such as Dossey’s (1995) *Healing Words: The Power of Prayer and the Practice of Medicine*, there is very little scientific evidence examining the effectiveness of prayer on survival for the individual who is praying (i.e., the petitioner).

### Prayer and Health

Prayer is often constructed as an ongoing interactive process involving individuals and a perceived divine other who may become an intimate member of their personal “social” network. Prayer may be associated with physical and mental health benefits through various mechanisms, ranging from the biological and physiological to the psychosocial and behavioral (for a review, see Koenig et al., 2012). From a clinical perspective, when working with individuals experiencing health difficulties, investigating their use of religious/spiritual practices such as prayer for coping may assist efforts to improve their overall wellness.

Due to greater interest in the potential benefits of prayer on health, researchers have developed various taxonomies of prayer (e.g., types, directions, expectancies) to investigate how this multidimensional and individualized phenomenon relates to physical and mental wellbeing. For example, Poloma and Pendleton (1991) identified four types of prayers (colloquial, petitionary, ritual, and meditative), whereas Laird et al. (2004) identified five (adoration, confession, reception, supplication, and thanksgiving). Foster’s (1992) focus on the interpersonal led to the identification of three directions of prayer (inward, upward, and outward), which Ladd and Spilka (2002) developed a measure to assess. Others yet have investigated expectations that the petitioner may hold around their prayers being heard and answered (e.g., Krause & Hayward, 2014; Upenieks, 2022). The study of such dimensions of prayer has certainly led to progress in the study of prayer and health. However, some of these classifications may be more relevant for certain groups than others (e.g., confession, meditation), limiting the generalizability of findings among larger and more diverse samples (e.g., the U.S.).

On the other hand, frequency of prayer may capture the use of prayer across its various dimensions, and may also be related to other aspects of religious/spiritual life such as religious service attendance or commitment to one’s faith, both of which are associated with lower mortality across religious groups (Koenig et al., 2012). Frequency of prayer may also be particularly relevant for individuals dealing with chronic illnesses, who may pray more as a means of coping with their difficulties (Barnes et al., 2002; Masters & Spielmans, 2007). Whether praying confers physical health benefits for individuals living with chronic illnesses may be assessed via the relationship between frequency of prayer and survival over time. However, in one of the few reviews looking exclusively at prayer and broader health-related outcomes, most of the studies reviewed (10/12) were cross-sectional and investigated intercessory prayer, many of the outcomes were for mental (rather than physical) health, and none assessed survival (Simão et al., 2016). Further, a perusal of books offering comprehensive reviews of the literature on medicine, religion, and health confirmed the lack of studies examining whether frequency of prayer predicts mortality (Koenig et al., 2012; Levin, 2020; Spilka & Ladd, 2013). Some (e.g., Helm et al., 2000) have examined frequency of prayer as part of private religious activities and found that a composite measure (including prayer, meditation, or bible study) predicted survival in 3,851 older adults (but not after controlling for demographic and health variables). However, prayer, meditation and bible reading are very different activities, and it is not clear which of these contributed to the findings.

Our review of the literature revealed only two studies investigating whether frequency of prayer longitudinally predicts survival in the petitioner. VanderWeele et al. (2017) found that praying several times per day was not associated with mortality in 36,613 black women followed over 8 years, although there was a trend toward *increased* mortality after controlling for other religious/spiritual variables. Another study controlling for initial disease status in people living with HIV (PLWH) found that frequency of prayer did not predict survival over 17 years, although praying for known others (vs. for oneself or for unknown others) was associated with greater survival for the petitioner. (Ironson & Ahmad, 2022). Given the well-established relationships between religious/spiritual variables and mortality (Chida et al., 2009; Koenig et al., 2012), further investigations are needed to determine whether frequency of prayer longitudinally predicts survival in individuals with chronic illnesses.

In their comprehensive review of prayer and health, Masters and Spielmans (2007) critiqued the literature and recommended that future studies investigating frequency of prayer and health should use larger samples and longitudinal methods that control for illness at earlier stages, and should specifically investigate individuals who have chronic illness. Thus, our study investigates whether frequency of prayer predicts survival over six years in a nationwide U.S. sample with chronic illness, after controlling for biomedical, sociodemographic, psychosocial, and health behavior variables at baseline.

## Method

### Participants

The data for the current study come from two waves of a nationwide (U.S.), face-to-face, random probability survey of people aged 18 and older (the Landmark Spirituality and Health Survey). Baseline interviews (Wave 1; *N* = 3,010) were conducted in 2014 by the National Opinion Research Center (NORC). Follow-up interviews (Wave 2) were conducted by Harris Insight in 2020. Of note, this was during the beginning of the COVID-19 pandemic and was also during an election cycle. All the independent variables and covariates in the analyses presented below come from the Wave 1 survey. For the purposes of this paper, Wave 2 data was only used for augmenting the death data. Thus, all participants were followed for at least six years and up to 7 years (from 2014 to 2020).

Since our main focus is for people who are medically ill, we restricted the analyses to people with at least one chronic illness (*n* = 1931) at the Wave 1 interview (in the previous 12 months) from a list of 12 chronic illnesses (e.g., cardiovascular disease; diabetes; cancer/malignant tumor; Liang, 1990) who also answered the prayer frequency question (*N* = 1,918). Participant demographics can be seen in Table 1.

**Table 1:** Demographic Characteristics for Participants with a Chronic Illness (n = 1931), with Prayer Data for those who also answered the Prayer Frequency Question (N = 1918)

Variable	%	*n*	Missing
Male	40.4%	780	0
Race/Ethnicity			11
African American	13.9%	269	-
Hispanic	11.6%	222	-
White	72.0%	1373	-
Health Behaviors			
Current Smoker (Yes)	40.6%	784	7
Drinks Alcohol (Yes)	50.2%	970	13
Alcohol (> 1 drink/day)	14.7%	284	13
Moderate Exercise (≥ 1/week)	75.5%	1,458	5
Body Mass Index > 28.4	50%	966	236
Prayer Frequency			
Did not pray	9.9%	190	
Less than once a month	4.3%	83	
Once a month	1.9%	36	
A few times a month	4.3%	83	
Once a week	2.5%	48	
A few times a week	11.2%	215	
Daily	24.5%	469	
More than once daily	41.4%	794	
Variable	*M*	*SD*	Missing
Age	58.69	17.74	37
Education in Years	13.32	3.25	1
Body Mass Index	29.47	6.70	236
Depression	6.22	2.72	8
Social Support	9.47	2.15	12

### Procedures

Death data was obtained for the Wave 1 participants using the National Death Index (NDI), maintained by the CDC, through December 31, 2020. The NDI uses identifying and demographic information to identify possible matching public death records (in this case 210 deaths). We supplemented this with informant reports of mortality identified during Wave 2 (six additional deaths). Finally, a search of obituaries, as well as voting records, and the internet using additional information (hobbies, nicknames), helped identify 16 deaths. After overlap, altogether we obtained data on 226 deaths. Mortality was coded as 1 = dead (i.e., died after Wave 1), and 0 = presumed alive. Restricting our sample to those who had a chronic illness yielded a sample with 213 deaths. Institutional review board approval was received from the University of Miami.

### Measures

#### Prayer

Frequency of private prayer was assessed by the question “How often do you pray by yourself?” (Fetzer, 1999). It was measured on a 1 to 8 scale (1 *=* Never, 2 = Less than once a month, 3 = Once a month, 4 = A few times a month, 5 = Once a week, 6 = a few times a week, 7 = Once a day, and 8 = Several times a day).

#### Control Variables

Biomedical control variables included age (in years) and physical health/symptoms at baseline (Wave 1). For physical symptoms, participants were asked whether they had each of 11 symptoms: pains in the heart or tightness or heaviness in the chest; trouble breathing or shortness of breath; swollen ankles; pains in the back or spine; repeated pains in the stomach; frequent headaches; constant coughing or frequent heavy chest colds; stiffness, swelling, or aching in any joint or muscle; getting very tired for a short time; dizziness or nausea; or frequent cramps in the legs (Magaziner et al., 1996). The symptom score was the number of symptoms they reported having.

Sociodemographic variables were sex (1 = men, 0 = women), race (1 = Black, 0 = White), and education in years.

Psychosocial variables included depression and social support. Depression was measured by 4 items (α = 0.85) from the CESD (Radloff, 1977) representing depressed affect (blues, depressed, crying spells, sad), asking how often you felt this way in the past week on a scale of 1 (Rarely/None of the time) to 4 (Most/All of the time). Social support included 3 items (α = 0.81) from Krause (2008) measuring how often social support was received from friends and family (e.g., How often do your family and friends express interest or concern for your well-being?). Each item was measured on a 1 (“Never”) to 4 (“Very Often”) scale.

Health Behaviors included: alcohol use, smoking, frequency of moderate exercise, and Body Mass Index (BMI). Alcohol use was the number of days the participant reported drinking (beer, wine or liquor) in the last month multiplied by the number of drinks consumed on the days they drank. Smoking was assessed by: “Do you smoke cigarettes now?” Moderate exercise was defined as activities like “fast walking, baseball, tennis, easy bicycling, volleyball, badminton, easy swimming, alpine skiing, or popular or folk dancing.” Participants were asked “How many days in the average week do you do at least 15 minutes of moderate exercise that does not make you feel exhausted?” (Krause et al., 2017).

### Statistical Analysis

Cox proportional hazards regression (using SPSS version 26) was used to determine whether frequency of private prayer predicted survival over 6 years among individuals with at least one chronic illness at baseline. Prediction to survival was evaluated after the effects of four covariate blocks of baseline measures were controlled statistically. These were chosen a priori to reflect variables correlated with both religion/spirituality and survival, noted as important in literature reviews, and variables consistent with our prior work (Masters & Spielmans, 2007; Powell et al., 2003). First, biomedical variables (age, baseline physical symptoms) were adjusted for in block 1. Sociodemographic variables (sex, education, and race) were adjusted for in block 2. Block 3 covariates, the psychosocial variables, included depression and social support. Block 4 included heath behaviors (alcohol use, smoking, moderate exercise, and BMI/weight). Frequency of prayer was entered thereafter. Listwise deletion procedures were used to deal with item non-response. As a result of the different covariate models, the number of cases for the Cox Regression analyses ranged from 1621 to 1881.

## Results

About two-thirds of the sample with a chronic illness reported praying either daily or more often (24.5% plus 41.4%). About 10% of our sample reported that they did not pray. The rest of the sample prayed a few times a week (11.1%), once a week (2.5%), a few times a month (4.9%), once a month (1.9%), or less than once a month (4.3%). Comparing this to the parent sample, those with chronic illness prayed significantly more than those without a chronic illness (*t* = 7.20, *p* < .001) with 60.3% of the parent sample praying daily or more often, and 12% saying they did not pray. This compares with national statistics (Pew, 2022) where 55% say they pray at least daily and 23% say they pray seldom or never (less than monthly).

### Preliminary Analyses

In terms of our covariates, higher prayer frequency was significantly correlated (all *p*’s < 0.01) with older age (0.102), more symptoms of physical illness (0.089), female gender (0.217), black race (0.134), less education, (-0.083), less alcohol use (-0.185), being overweight (BMI: 0.099), and greater social support (0.212). Praying was not significantly related to smoking, exercise or depression (*p*’s > 0.10).

### Survival Analyses

After six years of follow-up (2014 through 2020), 7.5% of the parent sample of 3,010 had died. Restricting the analysis to those with at least one chronic illness (*n* = 1931), the death rate was 11.2%. The results of the Cox regression survival analyses are presented in Table 2. The first row of Table 2 shows that frequency of prayer predicted significantly lower mortality (and greater survival) after controlling for biomedical variables (age and symptoms at baseline, which we consider to be minimal controls). As Table 2 also shows, greater frequency of prayer also predicted significantly lower mortality after additional controls for sociodemographic variables (line 2), psychosocial variables (line 3), and health behaviors (line 4). Note that comparing the model with only the biomedical controls to the prayer model with all control variables (-.092/-.065) reduced the *B* or “Hazard Rate function” by 31%. This, combined with the rest of Table 2, suggests that while sociodemographic, psychosocial, and health behavior variables accounted for a portion of the prayer-survival relationship, they did not account for enough to change the significance of the prayer-survival relationship.

**Table 2:** Results of Cox Regression with Prayer Predicting Mortality/Survival Adjusting for Biomedical Sociodemographic, Psychosocial and Health Behavior Variables in a Nationwide sample

	*B*	*SE*	𝜒^2^Δ Wald	*p*	HR – mortality (95% CI)	HR – survival (95% CI)
Prayer (biomedical) ^a^	− 0.092	0.028	11.023	0.001	0.912 (0.863-0.963)	1.096 (1.038–1.159)
Prayer (+ sociodemographic)^b^	− 0.082	0.029	8.133	0.004	0.921 (0.870-0.975)	1.086 (1.026–1.149)
Prayer (+ psychosocial) ^c^	− 0.079	0.029	7.154	0.007	0.924 (0.872-0.979)	1.082 (1.021–1.146)
Prayer (+ health behaviors)^d^	− 0.065	0.032	4.032	0.045	0.937 (0.879-0.998)	1.067 (1.002–1.138)
Prayer dichotomized ^a^	− 0.536	0.140	14.565	0.000	0.585 (0.445-0.771)	1.709 (1.297–2.247)
Prayer dichotomized ^d^	− 0.395	0.160	6.067	0.014	0.674 (0.492-0.923)	1.48 (1.083–2.033)

*Note. SE* = standard error; *df* = 1; Both HR-mortality and HR-survival are included for ease of interpretation

^a^Controlling for biomedical variables (age, and symptoms at baseline)

^b^Controlling for biomedical variables (age, and symptoms at baseline) and sociodemographic variables (sex, race, and education)

^c^Controlling for biomedical variables (above), sociodemographic variables (above) and psychosocial variables (depression, social support)

^d^Controlling for biomedical variables, sociodemographic variables, psychosocial variables, and health behaviors (alcohol, smoking, moderate exercise, and BMI)

**Fig. 1:**
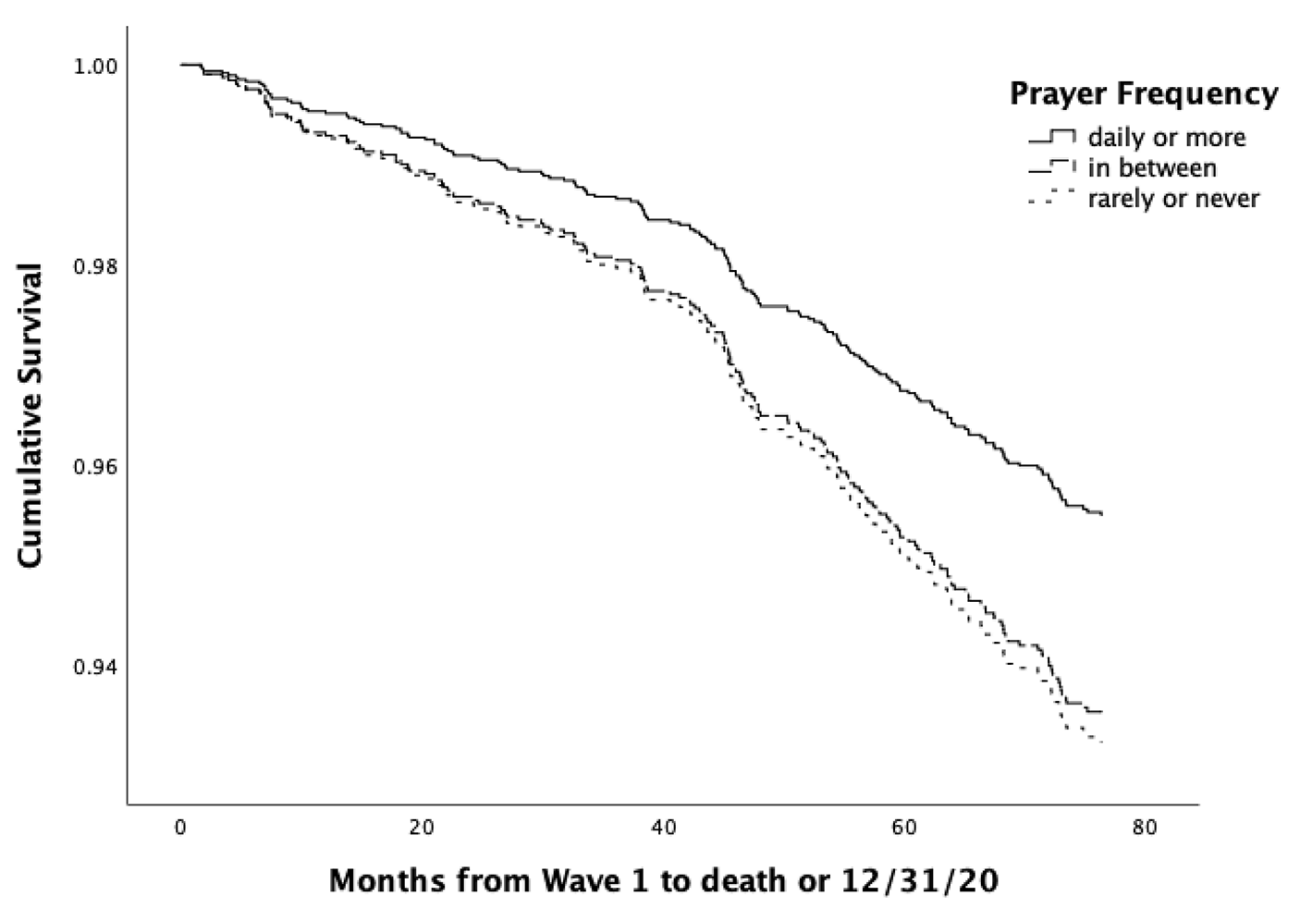
Survival Curves for Those who Prayed ≥ Daily vs. Those who Prayed Rarely/Never vs. Those in Between

For ease of interpretation, since the Hazard Ratio is dependent on the metric used for the prayer frequency variable, we also calculated the Hazard Ratios dichotomizing the prayer frequency variable (at the value closest to the median) into those praying daily or more versus those praying less than daily (see the bottom of Table 2). With minimal controls for only biomedical variables, those praying daily or more were 1.7 times more likely to survive over 6 years than those praying less often than daily. Using the most stringent controls for biomedical, sociodemographic, psychosocial and health behaviors, those praying daily or more were 1.5 times more likely to survive. Figure 1 depicts Survival Curves using Cox regression for those who prayed daily or more often vs. those who prayed rarely or never vs. those in between (i.e., once a month to a few times a week), controlling for biomedical variables (age, physical symptoms at baseline), sociodemographic variables (gender, education, and race/ethnicity), psychosocial variables (depression, social support) and health behaviors (alcohol, smoking, moderate exercise, weight [BMI]). The survival curve is clearly higher for those who pray more frequently.

## Discussion

We believe this is the first study to show that greater frequency of private prayer predicts survival. Although there are many studies on religion/spirituality and survival, little work has been done specifically in the area of prayers and survival for the petitioner, especially in the medically ill, and studies generally combine prayer with other spiritual practices, such as meditation. Thus, our study has several important design features: It is a nationwide sample, the subsample studied has at least one chronic illness, we controlled for a large variety of relevant covariates (most importantly baseline health, but also sociodemographics, psychosocial variables, and health behaviors), we examined frequency of prayer separately from other religion/spirituality variables, and this was a longitudinal study. We found that people with a chronic illness who prayed daily or more were 1.5 to 1.7 times more likely to survive (depending on covariates) over 6 years than those who prayed less often.

While the only other two studies looking specifically at prayer and survival did not find that frequency of prayer predicted survival, they were on very different samples: Black women (VanderWeele et al., 2017), and people with HIV (Ironson & Ahmad, 2022), whereas the present study has a nationwide sample. Our study also had different covariates: While all studies had multiple covariates, in terms of medical variables we controlled for symptoms present at baseline whereas the VanderWeele et al. (2017) study controlled for a history of cancer, MI, and stroke, and the Ironson and Ahmad (2022) study controlled for CD4 and Viral Load at entry.

Why might prayer predict better survival for the petitioner? Psychosocial, biological, and health behaviors provide possible pathways that have been reviewed at length by Koenig et al. (2012) and uncovered through recent studies. Although we controlled for many of the implicated mechanisms in this study, there are many others that we did not assess. Psychosocial pathways associated with greater religion/spirituality or praying include having a greater sense of peace, meaning/will to live, hope/optimism, greater social support, and less depression and anxiety (Ciarocchi et al., 2008; Ironson et al., 2002; Koenig, 2010; Krause, 2017; Powell et al., 2003; Schuster et al., 2001; Tataryn and Chochinov, 2002). In turn, these have been related to lower mortality (Chou et al., 2012; Czekierda et al., 2017; Giltay et al., 2004; Ironson et al., 2002, 2021; Ironson & Fitch, 2016; Park et al., 2016). Additional psychosocial pathways that may impact the effectiveness of prayer on health include one’s relationship with the divine. For example, people living with HIV who believed in a benevolent (vs. punishing) God had slower disease progression over 4 years (Ironson et al., 2011). As noted in the introduction, expectancy effects may also play a potential role between prayer and survival. People who pray likely expect (or at least hope) that God is listening to their prayers and will help them. Importantly, people who believe a medicine or procedure will work, even if it is inert, benefit from it as part of the well-known and documented placebo effect (e.g., Moseley et al., 2002).

Physiologically, people who pray more are more likely to have lower cortisol reactivity, blood pressure reactivity, and faster cardiovascular recovery in stress paradigms (Masters et al., 2004; Masters et al., 2020; Spilka and Ladd, 2013; Tartaro et al., 2005). Other biological pathways have linked psychosocial variables (including religion/spirituality) to various illnesses including cardiovascular disease, cancer, and HIV (Ironson et al., 2016; Ironson & Fitch, 2016; Mulder et al., 1992). These include sympathetic pathways activated during stress and their corresponding neurohormones (norepinephrine, epinephrine), the HPA axis (including cortisol), oxytocin, psychoneuroimmune pathways, inflammation/CRP, telomere length, and Conserved Transcriptional Response to Adversity (CRTA), which buffers against proinflammatory gene expression (Cole, 2019; Ironson et al., 2018; Kelsch et al., 2013; Mulder et al., 1992). Frequent prayer may alleviate the negative effects of allostatic load (i.e., the accumulation of physiological perturbations experienced as a result of daily stressors) through developing greater sense of peace and hope/optimism. In support of this, one national U.S. study (Bruce et al., 2017) found that allostatic load was lower in frequent church attenders and predicted survival over 18 years.

Turning to behaviors, some studies estimate that about half of the religion/spirituality – health relationship is due to healthier behaviors, such as less substance use, smoking, and moderate exercise (Hummer et al., 1999; Koenig et al., 2012; Krause, 2017; Oman et al., 2002).

### Limitations and Future Directions

One cannot establish causality from this or any other longitudinal study. Both frequency of prayer and survival may be caused by a third variable (e.g., conscientiousness). While we controlled for multiple covariates, there are others (e.g., dietary restriction, medication adherence). Furthermore, many other aspects of religion/spirituality remain to be studied. Notably, our models do not address the question of the intersectionality of prayer and religious service attendance. We believe this is an important question that will be addressed in future publications. In addition, investigating frequency of prayer alone has its limitations as the effects of prayer vary according to prayer types, directions, and expectancies (e.g., Krause and Hayward, 2014; Upenieks, 2022). Future studies may investigate how specific forms of prayer (e.g., for oneself vs. known others vs. unknown others) relate to survival in individuals with chronic illness, and not just frequency of prayer (e.g., Ahmad & Ironson, 2022). Further, other psychological variables (besides depression and social support) such as meaning in life and optimism may also explain the relationships we observed. Although our study consists of a nationwide sample and is thus somewhat generalizable, participants self-selected into the study, limiting external validity. Further, results may differ or have different implications with non-clinical samples, as those who are dealing with illness are more likely to pray in the first place. Importantly, this study does not address whether God or a supernatural force answers prayers.

### Clinical Implications

Physicians should be aware that many people use prayer for coping with illness. Spilka and Ladd (2013) conclude their chapter on Prayer and Health by saying that the psychological aspects of medical conditions may have the best potential for the utility of prayer, but our study suggests there may be benefits for survival as well. Ironson et al. (2016) suggest that one way to engage patients is to ask how they are coping with illness. If patients mention religion/spirituality, that provides an entry point for further discussion of broader aspects of using religion/spirituality (including prayer) to cope with chronic illness that are related to less depression, better quality of life, slower disease progression, and longer survival (Ironson et al., 2006; Lucette et al., 2016; Tsevat, 2006). Koenig (2013) and Kristeller et al. (2005) have also developed spiritual approaches and assessment that may be incorporated into patient care. Of relevance, a review of religion/spirituality literature showed an 18% reduction in mortality, and this compared more favorably than 60% of the reviewed health interventions (including statin therapy and fruit and vegetable consumption; Lucchetti et al., 2011). However, our findings in no way imply that prayer should be used as an alternative to good medical treatment; rather, prayer may complement other forms of needed treatments. As a cautionary note, using spiritual beliefs and practices to avoid engaging in psychological work (i.e., spiritual bypass) has been shown to reduce the benefits of religious coping on mental health (Ahmad et al., 2022). Whether incorporating our findings into clinical practice would result in improved medical outcomes remains to be determined. The authors hope that this study might spur interest and research on prayer as an adjunct to harness psychospiritual factors that have been used for thousands of years to be studied seriously in their potential role for facilitating the healing process.

## Data Availability

Although the data will not be made available at this time as we are embargoing the data until the research team has completed publishing from this project, it will be made available at a later time upon request. The output from this analysis is available upon request.
